# Measurement of corneal and limbal epithelial thickness by anterior segment optical coherence tomography and in vivo confocal microscopy

**DOI:** 10.1186/s12886-016-0342-x

**Published:** 2016-09-20

**Authors:** Qihua Le, Yan Chen, Yujing Yang, Jianjiang Xu

**Affiliations:** 1 Department of Ophthalmology, Eye & ENT Hospital of Fudan University, No. 83 Fenyang Road, Shanghai, 200031 China; 2 Research Center, Eye & ENT Hospital of Fudan University, Shanghai, 200031 China; 3 Myopia Key Laboratory of Ministry of Health, Eye & ENT Hospital of Fudan University, Shanghai, 200031 China

**Keywords:** Corneal epithelium, Limbal epithelium, Epithelial thickness, Anterior segment optical coherence tomography, In vivo confocal microscopy

## Abstract

**Background:**

To compare corneal epithelial thickness (CET) and limbal epithelial thickness (LET) measured by anterior segment optical coherence tomography (AS-OCT) and in vivo confocal microscope (IVCM) in normal subjects, and evaluate the consistency between them.

**Methods:**

Thirty-eight normal subjects (17 men and 21 women) were enrolled in this study. AS-OCT was performed at central cornea and the superior, inferior, nasal and temporal limbus. Then followed by IVCM examination performed at the same location. Agreement was analyzed by mean difference (AS-OCT minus IVCM), 95 % limits of agreement (LoA) (1.96 standard deviation of the difference), and Bland-Altman analysis.

**Results:**

The average CET measured by AS-OCT and IVCM was 55.6 ± 4.0 μm and 51.9 ± 4.9 μm respectively. The value measured by IVCM was significantly lower than that measured by AS-OCT (*P* = 0.015). The average LET values tested by AS-OCT were 10.3 and 10.9 % less at nasal and temporal quadrant (nasal: *P* = 0.019, temporal: *P* = 0.003), and were similar as those measured by IVCM at superior and inferior quadrant. In subjects older than 40 years, CET and LET values measured by AS-OCT were significantly higher than those by IVCM. Such differences were not found in subjects ≤ 40 years old.

**Conclusions:**

CET values measured by IVCM are lower than those by AS-OCT, while LET values measured by two devices have good agreement. These two techniques have their own advantages in measuring epithelial thickness and are mutually complementary.

## Background

Corneal epithelium plays a crucial role in maintaining the integrity and function of cornea. Limbus, the transitional zone between the cornea and the bulbar conjunctiva, is believed to be the harbor of corneal stem cells and essential for self-renewal and metabolism of corneal epithelium [1]. The alterations of corneal epithelial thickness (CET) are found in many pathological conditions such as contact lens wear [2], keratoconus [3], and disorders implicating limbal stem cell deficiency (LSCD) [4]. Recent studies confirm that aging and other pathological conditions could lead to limbal epithelial thinning [4,5], indicating that the change of corneal and limbal epithelial thickness (LET) is an indicator of corneal structural or functional alterations.

A number of methods are currently available for epithelium thickness measurements, including anterior segment optical coherence tomography (AS-OCT) [2,3,5], scanning ultrasonic biomicroscopy [6], very high-frequency digital ultrasound [7], and in vivo confocal microscope (IVCM) [4,8]. Among them, Fourier domain AS-OCT has been the most widely-used equipment to measure CET and LET in the recent years. This non-contact technique provides a repeatable and reproducible way to measure CET and LET with the advantages such as higher scanning speed and less demand for cooperation from the patients. However, AS-OCT system cannot discriminate tear film at present due to the limitation of resolution. Even though Werkmeister and coauthors measured the thickness of precorneal tear film using a custom-built ultrahigh-resolution AS-OCT [9], it has not been commercially available heretofore. Another imaging technique, IVCM, provides the measurement of epithelial thickness through calculating the distance between the depth of corneal superficial epithelial cells and basal epithelial cells. Although the way of measurement facilitates the exclusion of the impact on epithelial thickness caused by tear film, IVCM requires contact between a probe and the ocular tissues, which might cause interference to the measurement more or less.

To the best of our knowledge, no reports have been published yet to determine the agreement of AS-OCT and IVCM concerning the measurement of CET and LET. However, we consider that this issue is important in the interpretation of epithelial thickness because these two devices are widely-used in the clinic. Therefore, we perform this study to compare CET and LET measured by AS-OCT and IVCM in normal subjects and evaluate the agreement between them.

## Methods

### Study subjects

A total of 38 normal subjects (17 men and 21 women) were enrolled in this study, with an average age of 43.1 ± 13.8 years (range: 22–68 years). Before enrollment, all subjects underwent routine ocular examinations to exclude ocular abnormalities except ametropia. The subjects with definite diagnosis of diabetes mellitus or autoimmune diseases were also excluded. Only the right eye of each subject was chosen for AS-OCT and IVCM examination. The study was approved by the Ethics Committee of Eye, Ear, Nose and Throat Hospital of Fudan University and was carried out in accordance with The Declaration of Helsinki. Written informed consent was obtained from all the participants.

### AS-OCT and measurement of epithelial thickness

AS-OCT was performed before IVCM so as to avoid potential artifacts or interference caused by direct contact between probe and ocular tissues. Fourier-domain OCT system (RTVue-100; Optovue Inc., Fremont, CA, USA) with a cornea anterior module long adapter lens (1.96-mm scan depth and 6-mm scan width) was used in this study. The device worked at 830-nm wavelength and had a scan speed of 26,000 axial scans per second. The axial resolution of the system was 3 μm. To obtain the images of central cornea, pachymetry scan mode was selected and the scans were centered on the coaxially fixating corneal light reflex identified by the central bright reflection on the OCT scan. To obtain the images of limbal area in each quadrant (superior, inferior, nasal, and temporal), cross-line scan mode was used and the subject was asked to fixate at a peripheral target to maintain the perpendicularity of the OCT beam at the surface of the targeted tissue, which was essential for obtaining accurate thickness values. All tests were performed by one trained operator.

**Fig. 1:**
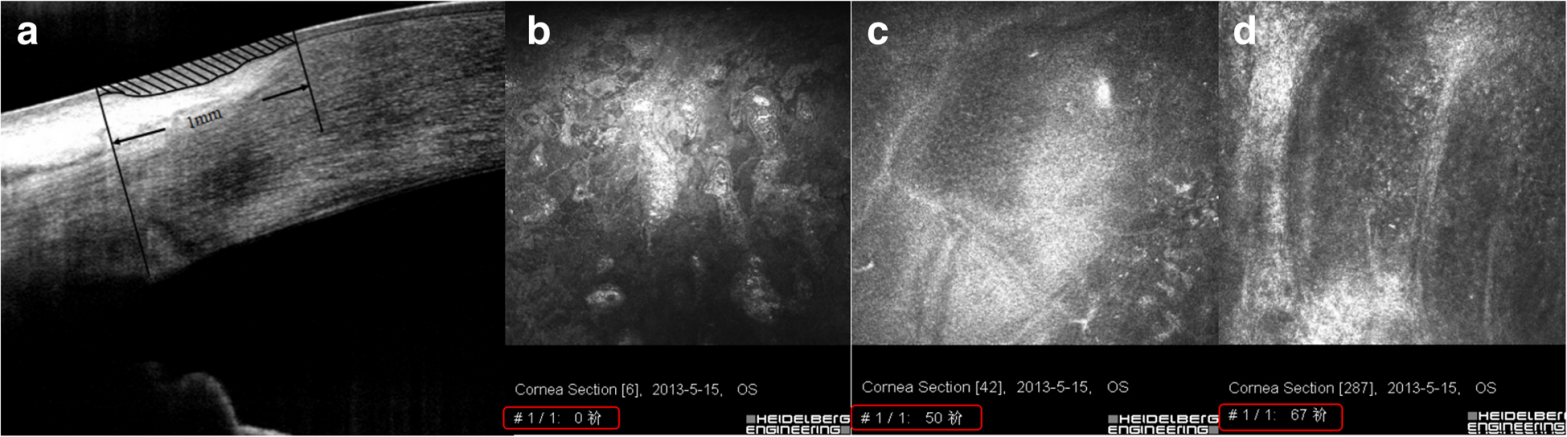
Measurement of epithelial thickness by AS-OCT (**a**) and IVCM (**b**-**d**). **a** Evaluation of limbal epithelium in an AS-OCT cross-line scan image. The corneoscleral transitional zone was firstly determined according to the limbus anatomical landmarks, which was 1.0-mm wide extending centripetally from the scleral spur according to the anatomical definition. Shaded region indicates the target limbal epithelium. The LET was measured between anterior and posterior epithelial surface (*two black curves*). **b** The image of corneal superficial epithelial cells. The depth of superficial of epthelium was 0 μm, as shown in the red frame. **c** The final image of basal epithelial cells at central cornea. Its depth was 50 μm. **d** The final of image of basal epithelial cells between the palisades of Vogt at limbal area. Its depth was 67 μm

The measurement of CET and LET was performed by an experienced ophthalmologist, who was blind to the subjects’ demographic features. The epithelial thickness map was automatically generated by the built-in software of AS-OCT (version A6.9.0.27, Optovue, Inc.) [9,10]. The thickness of epithelium in central 2-mm diameter zone was obtained as CET. Before the measurement of LET, a corneoscleral transitional zone was firstly determined, which was usually 1.0-mm wide extending centripetally from the scleral spur according to the anatomical definition, just as shown in Fig. 1a. Then mean LET in this area was measured with the software (Image-Pro Plus; Media Cybernetics, Inc., Rockville, MD, USA) as we previously reported [5]. Both CET and LET were determined as the average of 3 independent measurements.

### In vivo confocal microscopy analysis

The Heidelberg Retina Tomograph III Rostock Corneal Module confocal microscope (Heidelberg Engineering GmBH, Dossenheim, Germany) was used in the study and Z-scan images were taken by a trained technician. At first, the central cornea of the enrolled eye was examined. Then the subjects were required to look downward, upward, to the left side and to the right side to examine the superior, inferior, nasal and temporal limbus. At each limbal area, three examination points were selected: the location where the Vogt of Palisades were found (central location), 0.5 mm centripetal to corneal center from central location, and 0.5 mm centrifugal to corneal center from central location. At least three volume scans were collected at every examination point for each eye. The volume scans with minimal motion artifacts were selected for analysis. Epithelial thickness was obtained by manual counting the depth of focus position from the initial image of the superficial epithelium to the final image of the basal cell layer just as previously described [4]. In brief, when POV were found at limbus, we slowly moved the focus deeper to check the images of basal epithelium between the adjacent two palisades on the monitor until the hyporeflective basal epithelial cells were replaced by the hyperreflective stromal cells. The depth of the last image in which basal epithelium could be discerned was used for the measurement of limbal epithelium (Fig. 1b-d). The average thickness of three examination points measured at each limbal area was considered as LET.

### Statistical analysis

Data were statistically analyzed by SPSS program statistical package V13.0 (SPSS Inc, Chicago, IL, USA). Basic descriptive statistics were calculated on all data gathered, and values were reported as mean ± SD. Difference (bias) in epithelial thickness values for each area was calculated as AS-OCT minus IVCM. A negative difference indicated a thinner value on AS-OCT compared to IVCM. Normality of data was determined first using Shapiro-Wilk Test. A paired *t* test was used to analyze the data conforming to Gaussian distribution, and Wilcoxon Signed Ranks Test was applied for data with nonnormal distribution. The agreement between both devices was determined using Bland–Altman analysis, and was verified by cross classification and interclass correlation coefficient (ICC) analysis.. The mean and 95 % limits of agreement(LoA) (1.96 × standard deviation) of the bias were calculated for central cornea and each quadrant. A *P* value less than 0.05 was considered as statistically significant.

## Results

**Fig. 2:**
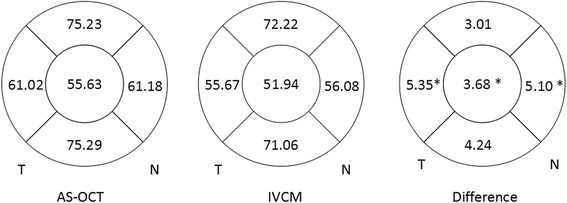
Schematic corneas displaying epithelial thickness of central, superior, inferior, temporal and nasal corneas. The figs. in each zone represented mean epithelial thickness measured by AS-OCT and IVCM and the bias (mean difference) between AS-OCT and IVCM (AS-OCT minus CM). An asterisk denoted significant difference in mean thickness reading for this epithelial zone between AS-OCT and IVCM (*P* <0 .05). T = temporal; *N* = nasal

**Fig. 3:**
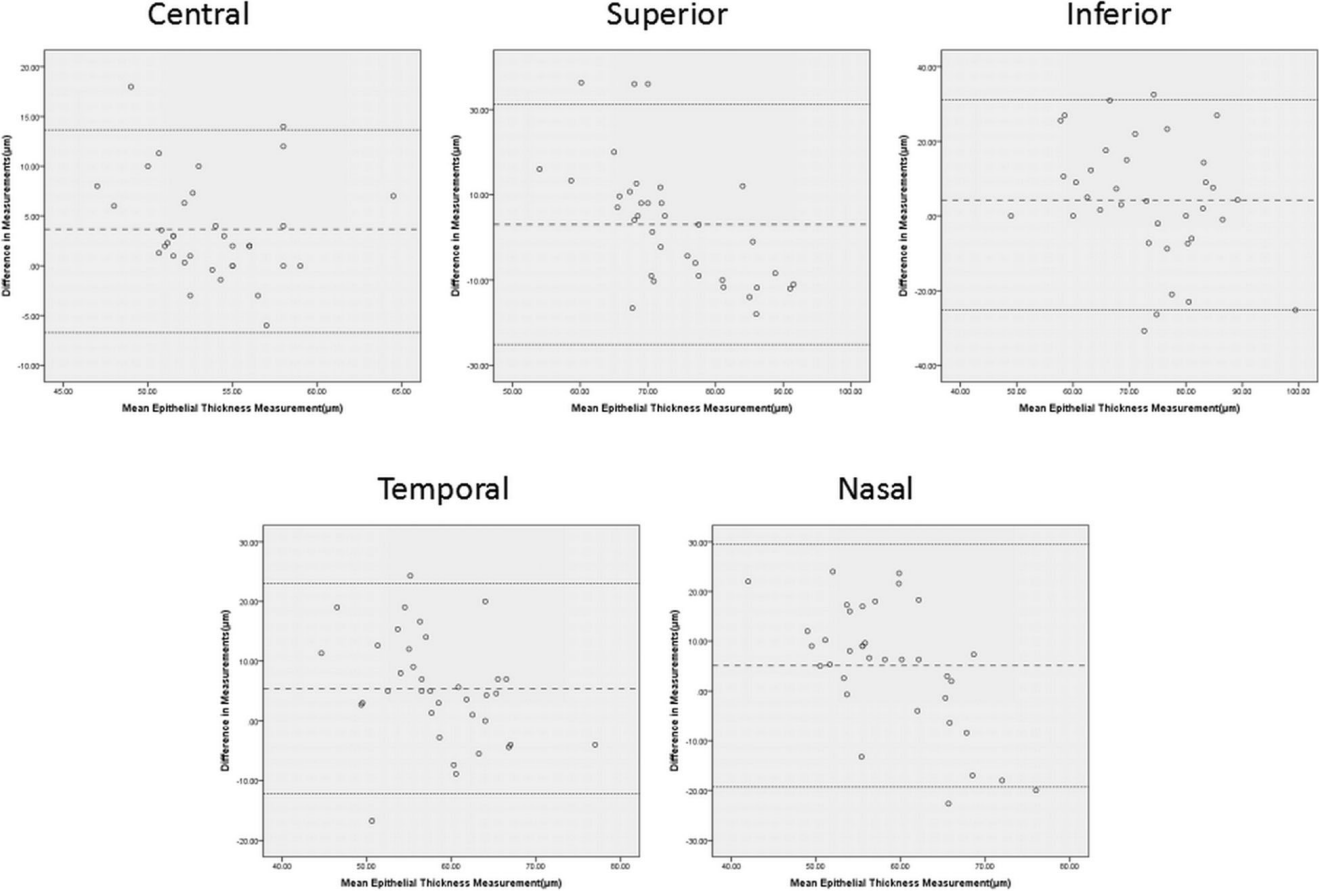
Bland-Altman charts showed difference in average corneal epithelial thickness measurements in relation to mean measurement between the two methods (AS-OCT minus IVCM). A positive difference showed that the values measured by AS-OCT was thicker. Bias ± 95 % limits of agreement were also displayed. No relationship was found between differences in central, superior, inferior, temporal or nasal epithelial thickness

The average CET measured by AS-OCT and IVCM was 55.6 ± 4.0 μm and 51.9 ± 4.9 μm respectively. The value measured by IVCM was significantly lower than that measured by AS-OCT (*P* = 0.015). The average LET values tested by AS-OCT and IVCM were shown in Fig. 2. The LET values measured by AS-OCT at nasal and temporal quadrant were significantly higher than those by IVCM (nasal: *P* = 0.019, temporal: *P* = 0.003). The LET values at superior and inferior quadrant were similar between AS-OCT and IVCM. Bland-Altman plots charts were presented in Fig. 3, which showed the difference in epithelial thickness at central cornea and each limbal quadrant between two devices. A positive difference showed that the values measured by AS-OCT was thicker. Bias ± 95 % limits of agreement are also displayed in Fig. 3. The evaluation of consistency between two devices was presented in Table 1.

**Table 1:** The evaluation on consistency between two devices

	Bias (μm)	95 % Limits of Agreement (μm)	Plots out of 95%LoA	Maximum Bias/Average
CC	3.68 ± 5.08	−6.27 ~ 13.64	5.56 %(2/36)	33.47 %
SL	3.01 ± 14.36	−25.17 ~ 31.19	8.33 %(3/36)	49.25 %
IL	4.24 ± 16.34	−27.79 ~ 36.27	8.33 %(3/36)	44.55 %
TL	5.35 ± 8.95	−12.27 ~ 22.97	5.56 %(2/36)	41.65 %
NL	5.10 ± 12.45	−19.30 ~ 29.50	5.56 %(2/36)	40.93 %

*CC* central cornea, *SL* superior limbus, *IL* inferior limbus, *TL* temporal limbus, *NL* nasal limbus

Concerning the thickness of corneal epithelium and limbal epithelium had a close relationship with age, the CET and LET values were further compared between two subgroups divided according to the age. Twenty people(9 men and 11 women) were older than 40 years old, and the rest 18 people (9 men and 9 women) were younger than 40 years. The gender distribution were similar between two groups (*P* = 0.877). Table 1 showed the result of comparisons within two subgroups. The CET and LET values examined by AS-OCT were similar to those by IVCM in subjects ≤ 40 years old. However, in subjects older than 40 years, the CET and LET values measured by AS-OCT were significantly higher than those by IVCM. Comparisons on CET and LET measured with AS-OCT between subjects younger than 40 and older than 40 didn’t show any statistically significant differences. The results were similar concerning those measured by IVCM, except that superior limbal epithelium was thicker in younger participants than older ones (*P* = 0.044), as Table 2 presented.

**Table 2:** CET and LET comparisons between subgroups ≤40 and >40 years old

		CC (μm)	SL(μm)	IL(μm)	TL(μm)	NL(μm)
≤40	IVCM	52.2 ± 4.4	77.2 ± 13.6	74.6 ± 14.5	58.2 ± 9.5	58.9 ± 13.5
	AS-OCT	54.9 ± 4.0	73.6 ± 6.0	76.1 ± 9.9	61.8 ± 7.6	60.0 ± 6.9
	P	0.282	0.064^a^	0.701	0.346	0.725
>40	IVCM	51.6 ± 5.2	67.3 ± 14.7	67.6 ± 14.9	53.2 ± 8.6	52.3 ± 10.5
	AS-OCT	56.3 ± 3.8	76.9 ± 8.1	74.5 ± 12.5	60.3 ± 5.6	62.4 ± 4.9
	P	0.006†	0.005†	0.007†	0.001†	0.003†
IVCM	≤40	52.2 ± 4.4	77.2 ± 13.6	74.6 ± 14.5	58.2 ± 9.5	58.9 ± 13.5
	>40	51.6 ± 5.2	67.3 ± 14.7	67.6 ± 14.9	53.2 ± 8.6	52.3 ± 10.5
	P	0.731	0.044*	0.077	0.104	0.165
AS-OCT	≤40	54.9 ± 4.0	73.6 ± 6.0	76.1 ± 9.9	61.8 ± 7.6	60.0 ± 6.9
	>40	56.3 ± 3.8	76.9 ± 8.1	74.5 ± 12.5	60.3 ± 5.6	62.4 ± 4.9
	P	0.28	0.178	0.656	0.511	0.235

*CC* central cornea, *SL* superior limbus, *IL* inferior limbus, *TL* temporal limbus, *NL* nasal limbus

^a^ Wilcoxon Signed Ranks Test was used because this paired group didn’t conform to Gaussian distribution, * *P* < 0.05, † *P* < 0.01, ‡ *P* < 0.001

## Discussion

Both AS-OCT and IVCM could be applied in the measurement of epithelial thickness, although the measurement principle of these two techniques were different. AS-OCT differentiated epithelium layer with substantia propria based on the different reflectivity at the interfaces due to refractive index changes. However, the measurement of IVCM used superficial epithelial cells as starting point and basal epithelial cells as the ending point in Z-scan mode. Since the differentiation of cellular structure was required in the measurement by IVCM, it had higher demand for the image acquisition and interpretation than AS-OCT during the process of examination.

It was not surprising that CET values measured by AS-OCT were approximately 4 μm thicker than those by IVCM in the present study. As we mentioned, the currently commercially available AS-OCT system was unable to discriminate the thickness of precorneal tear film with routine scan mode due to the limitation of resolution. The thickness of precorneal tear film was reported to be 3–5 μm [11]. Therefore, CET measured by AS-OCT and IVCM had good consistency under the condition that the thickness of tear film was deducted in the values taken by AS-OCT.

The comparisons on limbal epithelial thickness measured by AS-OCT and IVCM showed that they didn’t have statistically significant differences at superior and inferior quadrants. It indicated that these two techniques had good consistency in measuring superior and inferior LET. However, we found that limbal epithelium at nasal and temporal quadrant measured by AS-OCT were significantly thicker than those by IVCM. We supposed that it might be attributed to the differences in the principle of measurement. The structural observation at cellular level was not involved in the measurement by AS-OCT. Nevertheless, IVCM discriminated superficial epithelial cells and basal epithelial cells around the Vogt of Palisades to calculate epithelial thickness. It had been demonstrated that atrophy or absence of the Vogt of Palisades were more likely to be found at nasal and temporal quadrants than superior and inferior sides [12,13], which might cause interference to the recognition of limbal basal epithelial cells.

The comparison between younger participants and older ones revealed that the measurement of AS-OCT and IVCM had good consistency in subjects younger than 40-year-old. However, the corneal epithelium was significantly thinner measured by IVCM than AS-OCT in those older than 40. It had been reported that the thickness of corneal epithelial basement membrane increased while aging, especially at peripheral cornea [14–16]. AS-OCT discriminated epithelium layer with the basement membrane as the anatomical hallmark, while IVCM did not. It might be a possible explanation. It has been reported that LET at nasal and temporal quadrants decreased while aging [5]. However, the current study didn’t give similar findings. The small sample size of the current study might be the major reason. This issue would be addressed by further studies with a larger number of subjects.

A previous study reported that measurements of corneal sublayer thickness with confocal microscope showed poor repeatability when examining the thinner corneal layers, such as the epithelial layer [8]. However, our study found that with proficient manipulation from the technician, good cooperation from patients and accurate interpretation of acquired images from an experienced ophthalmologist, the measurement of corneal epithelium thickness with AS-OCT and IVCM had a satisfactory consistency. Moreover, IVCM could exclude the impact of tear film on the measurement, thus obtaining a more accurate value. Nevertheless, AS-OCT could measure the thickness at any point of epithelium layer in a cross-sectional scan, while IVCM only measure the epithelium thickness at the point where the examination was performed. Therefore, these two techniques were mutually complementary and couldn’t be replaced with each other.

Two limitations should be addressed in the current study. One is the small sample size. It hinders the detailed analysis on the age-related changes of epithelial thickness. Further studies with a larger number of subjects and age-stratified groups will be helpful to address this issue. The other is the variation of anatomy at limbal area. Limbus is a region transiting from cornea to sclera, with the width of 1-1.5 mm. The average thickness of limbal epithelium has a close relationship with the width of limbus because of its undulating features in the anatomy. Moreover, the range of LET in normal subjects has not yet been established because the already-published studies on LET were performed in different races and with various measuring methods. So large scale studies including subjects with different age and races are required in the future to establish the normal range of LET. It is of great importance to determine the changes of LET under pathological conditions.

## Conclusion

CET values measured by IVCM are lower than those by AS-OCT, while LET values have good consistency between them. These two techniques have their own advantages and are mutually complementary.

## Abbreviations

AS-OCT: Anterior segment optical coherence tomography
CET: Corneal epithelial thickness
IVCM: In vivo confocal microscope
LET: Limbal epithelial thickness
LoA: Limits of agreement
